# Protective effect of *Momordica charantia* water extract against liver injury in restraint-stressed mice and the underlying mechanism

**DOI:** 10.1080/16546628.2017.1348864

**Published:** 2017-07-13

**Authors:** Yuanyuan Deng, Qin Tang, Yan Zhang, Ruifen Zhang, Zhencheng Wei, Xiaojun Tang, Mingwei Zhang

**Affiliations:** ^a^ Key Laboratory of Functional Foods, Ministry of Agriculture, Guangdong Key Laboratory of Agricultural Products Processing, Sericultural & Agri-food Research Institute Guangdong Academy of Agricultural Sciences, Guangzhou, P. R. China

**Keywords:** *Momordica charantia*, restraint stress, liver function, antioxidation, mitochondria

## Abstract

**Background**: *Momordica charantia* is used in China for its *jianghuo* (heat-clearing and detoxifying) effects. The concept of *shanghuo* (the antonym of *jianghuo*, excessive internal heat) in traditional Chinese medicine is considered a type of stress response of the body. The stress process involves internal organs, especially the liver.

**Objective**: We hypothesized that *Momordica charantia* water extract (MWE) has a hepatoprotective effect and can protect the body from stress. The aim of this study was to investigate the possible effects of MWE against liver injury in restraint-stressed mice.

**Design**: The mice were intragastrically administered with MWE (250, 500 and 750 mg/kg bw) daily for 7 days. The Normal Control (NC) and Model groups were administered distilled water. A positive control group was intragastrically administered vitamin C 250 mg/kg bw. After the last administration, mice were restrained for 20 h.

**Results**: MWE reduced the serum AST and ALT, reduced the NO content and the protein expression level of iNOSin the liver; significantly reduced the mitochondrial ROS content, increased the mitochondrial membrane potential and the activities of mitochondrial respiratory chain complexes I and II in restraint-stressed mice.

**Conclusions**: The results indicate that MWE has a protective effect against liver injury in restraint-stressed mice.

**Abbreviations**: MWE: *Momordica charantia* water extract; *M. charantia: Momordica charantia* L.; ROS: reactive oxygen species; NO: nitric oxide; iNOS: inducible nitric oxide synthase; IL-1β: interleukin-1 beta; TNF-α: tumor necrosis factor alpha; IL-6: interleukin 6; IFN-γ: interferon gamma; VC: vitamin C; ALT: alanine transaminase; AST: aspartate aminotransferase; GSH: glutathione; GSH-PX: glutathione peroxidase; MDA: malondialdehyde; BCA: bicinchoninic acid; TBARS: thiobarbituric acid reactive substances; Trolox: 6-hydroxy-2,5,7,8-tetramethylchroman-2-carboxylic acid; JC-B: Janus Green B; DW: dry weight; FC: Folin–Ciocalteu; GAE: gallic acid equivalents; bw: body weight; NC: normal control group; Model: restraint stress model group; VC: positive control vitamin C group, 250 mg/kg bw; MWEL: *Momordica charantia* water extract low-dose group, 250 mg/kg bw; MWEM: *Momordica charantia* water extract middle-dose group, 500 mg/kg bw; MWEH: *Momordica charantia* water extract high-dose group, 750 mg/kg bw; HE: hematoxylin and eosin; ORAC: total oxygen radical absorbance capacity; ABAP: dihydrochloride; ATP: adenosine triphosphate

## Introduction

*Momordica charantia* L. is a typical sub-tropical vegetable. Many studies have proven that *M. charantia* contains active substances such as saponin, polysaccharide, protein and peptide, which possess hypoglycemic, lipid-lowering, anti-oxidative and anticancer biological activities [1–4]. In China, *M. charantia* is often dried into *M. charantia* tea (teabag and herbal tea). *M. charantia* tea extracted or brewed in hot water to drink is claimed to possess anti-diabetic, weight loss and *jianghuo* (literally, decreasing the internal heat) effects.

The concept of *shanghuo* (literally, antonym of *jianghuo*, excessive internal heat) in traditional Chinese medicine is connected to that of the stress response in modern medicine. *Shanghuo* is considered a type of response to a psychological and physiological stress load. It is a manifestation of physical and mental fatigue that is beyond the physiological regulation range. Stress is a nonspecific reaction of the body caused by stimulation from internal and external environments. The liver is the main executor of the stress response system [5]. Previous studies have demonstrated that restraint stress can increase the activity of serum transaminase and liver nitric oxide (NO) content [6,7]. Moreover, restraint stress can cause destruction of the mitochondrial structure in mice, the production of large amounts of mitochondrial reactive oxygen species (ROS), dysfunction of mitochondrial respiratory chain complexes, a decrease in the activities of antioxidant enzymes in the body, and a compromised anti-oxidative protection system of mitochondria in the liver, which in turn causes liver cell injury [8]. He et al. set up stress-models of mice to imitate *Shanghuo* and studied the therapeutic effect of Guangdong Herbal Tea (GHT) on *jianghuo*. They found the GHT protected against liver injury induced by restraint stress. The anti-stress mechanism of GHT was related to the protection effect against oxidative stress in a stress-loaded organism [5].

There have been many studies on hypoglycemic [9] and reduced adiposity [10,11] properties of *M. charantia*, but few on *jianghuo* effects. The mechanism of action and the material foundation about it are not known. In the present study, we prepared the *M. charantia* water extract (MWE) according to the daily drinking method and examined the protective effect of MWE against liver injury in restraint-stressed mice and to investigate the mechanism of the protective effect of MWE on the liver in terms of oxidative stress and mitochondrial structure and function. This is the first investigation to evaluate the effect of *M. charantia* on *jianghuo* effects.

## Material and methods

### Chemicals

Vitamin C (VC) tablets were purchased from Sanjing Pharmaceutical Co. Ltd. (Harbin, China). Trolox, fluorescein disodium salt and 2,2′-azobis (2-amidinopropane) dihydrochloride (ABAP) were purchased from Sigma-Aldrich (St. Louis, MO, USA). A mitochondria extraction kit and mitochondrial membrane potential assay kits were purchased from Beyotime Institute of Biotechnology (Guangzhou, China). Janus Green B (JC-B), a mitochondrial ROS fluorescence detection assay kit, a mitochondrial respiratory chain complex I kit and a mitochondrial respiratory chain complex II kit were all purchased from Shanghai GenMed Scientifics Inc. (Shanghai, China). Goat anti-rabbit immunoglobulin G (IgG) and an electrochemiluminescence (ECL) kit were purchased from Multisciences (Hangzhou, China). Rabbit anti-inducible iNOS (inducible nitric oxide synthase) antibody was purchased from Cell Signaling Technology Inc. (Boston, USA). The anti-mouse β-actin (1:2000) antibody was purchased from Santa Cruz Biotechnology Inc. (Dallas, Texas, USA). All other reagents were made in China and were analytically pure.

### Materials

*M. charantia* (variety: Lvbaoshi) was provided by the Vegetable Research Institute Guangdong Academy of Agricultural Sciences. Green and fresh *M. charantia* fruits were sliced into 3–5 cm sections after washing them and removing the seeds. Afterwards, the *M. charantia* slices were dried at 65°C for 20 h. The slices were crushed into powder using a 40-mesh sieve. The *M. charantia* powder was mixed with water at a solvent ratio of 1:15, and the mixture was then boiled and extracted for 2 h. The extract was filtered through a 100-mesh filter screen. Next, the filter residue was again extracted. The two filtrates were merged and concentrated in a vacuum rotary evaporator (Eyela N-1100, Eyela, Tokyo, Japan) at 55°C. The extract was then vacuum freeze-dried in a vacuum freeze dryer (FDU-2110, Eyela, Tokyo, Japan). The MWE powder was then stored at −20°C for further use.

### Determination of the main components in the MWE

The main components in the MWE are shown in Table 1. The phenol-sulfuric acid method [12] was used to determine the total polysaccharide content in the MWE, which was 27.92 g/100 g extract. Monosaccharide composition of the polysaccharides was performed using gas chromatography-mass spectrometry according to our previous work [13]. The vanillin-perchloric acid method [14] was used to determine the total saponin content in the MWE, which was 0.48 g/100 g extract. Saponin composition was performed using HPLC [15]. Saponin compounds were provided by Professor Minghua Qiu, Kunming Institute of Botany, Chinese Academy of Sciences. The purity of compounds was detected using HPLC as follows: Momorcharaside A, 95.759%;Momordicoside A,97.728%;Karaviloside XI,58.086%;Momordicoside F2,86.041%; Momordicoside K, 85.729%; Kuguacin N, 94.918%; (23*E*)-3*β*,7*β*,25-trihydroxycucubita-5,23-dien-19-al,97.976%. The national standards GB 5009.5–2010 was used to determine the total protein content in the MWE, which was 15.80 g/100 g extract. Protein composition was performed using GB/T 5009.124–2003. The Folin–Ciocalteu (FC) colorimetric method [16] was used to determine the total phenolic content in the MWE, which was 1.17 g gallic acid equivalents (GAE)/100 g extract. Phenolic composition was performed using HPLC according to our previous work [17].

**Table 1.** Main chemical compounds of *Momordica charantia* water extract.

	Compounds	Content
Polysaccharide (g/100 g)		27.92 ± 0.58
Protein (g/100 g)		15.80 ± 0.18
Saponin composition (μg/g)	Momordicoside A	4.82 ± 0.09
	Momorcharaside A	5.11 ± 0.06
	Kuguacin N	0.34 ± 0.03
	Momordicoside F2	58.62 ± 0.64
	Momordicoside K	101.24 ± 0.82
	Karaviloside XI	0.22 ± 0.03
	(23*E*)-3*β*,7*β*,25-trihydroxycucubita-5,23-dien-19-al	23.15 ± 0.42
Phenolic composition (mg/100 g)	Vanillic acid	39.18 ± 2.50
	Epicatechin	388.63 ± 9.30
	Rutin	27.88 ± 3.18

Values are the means ± *SD* (*n* = 3).

### Experimental animals and grouping

Seventy-two 6-week-old male Kunming mice, purchased from the Laboratory Animal Center of Southern Medical University (certificate number: SCXK (Guangdong) 2011–0015), were used as the experimental animals. The mice were raised at 25 ± 2°C, and the illumination period was 12 h/d (08:00–20:00). The experiment was conducted after 1 w of adaptive breeding. The experiment was approved by the Animal Care and Use Committee of Guangdong Province (Guangzhou, China) and performed according to the Laboratory Animal Management Regulations of Guangdong Province. The 72 mice were randomized into a normal control group (NC), a restraint stress model group (Model), a positive control vitamin C group (VC, 250 mg/kg body weight [bw]), a MWE low-dose group (MWEL, 250 mg/kg bw), a MWE middle-dose group (MWEM, 500 mg/kg bw) and a MWE high-dose group (MWEH, 750 mg/kg bw). There were 12 mice in each group.

The MWE and VC tablets were dissolved in distilled water according to the aforementioned doses. The MWE and VC solutions were prepared freshly before use. The mice in the experimental groups were intragastrically administered 0.1 mL/10 g bw of MWE every day according to the aforementioned doses for 7 days. The NC and Model groups were intragastrically administered 0.1 mL/10 g bw of distilled water instead. A positive control group was intragastrically administrated VC 250 mg/kg body weight. All the mice were fed (ad libitum) on normal rodent chow during the whole experiment. After the groups were intragastrically administered for the last time on the 7^th^ day, the mice in MWE and Model group were placed in 50 mL plastic centrifuge tubes to restrain for 20 h (12:30–08:30). Thirty minutes after restraining, the mice were anesthetized using ether. Blood samples were collected from the mice’s hearts. After the blood samples were centrifuged at 3000 r/min and 4°C in a centrifuge (Sorvall Biofuge Stratos, Thermo Electron, USA), the supernatant serums were collected. The liver of each mouse was collected by dissection. All samples were stored in a refrigerator at −20°C.

### Hepatic pathological structure

The fresh liver tissue was fixed in a 10% formalin buffer solution and embedded with paraffin. Tissue sections were randomly stained with hematoxylin and eosin (HE). The tissue sections were observed and photographed under an inverted fluorescence microscope (DMI3000 B, Leica, Germany) [18].

### Biochemical analysis of serum and liver homogenate

AST (glutamic oxaloacetic transaminase) and ALT (glutamic pyruvic transaminase) activities in the serum were determined spectrophotometrically using test kits according to the manufacturer’s instructions. The liver tissue from each mouse was added to nine times its volume of ice-cold physiological saline and was then mechanically homogenized in a blender (DS-1, Shanghai Specimen and Model Factory, China) in an ice bath, after which the homogenate was centrifuged at 2500 rpm for 10 min at 4°C. Bradford’s method was used to determine the protein content in the liver tissue homogenate. The GSH-PX and iNOS activity and GSH and NO contents in the liver tissue were measured with the corresponding commercial kits. The thiobarbituric acid reactive substances (TBARS) method was used to determine the liver and serum lipid peroxide concentrations (unit: nmol malondialdehyde [MDA] equivalents per mL serum or per mg protein) according to the instructions for the test kit.

The total oxygen radical absorbance capacity (ORAC) was determined previously as described by Ou et al. [19]: 200 μL of 0.96 μmol/L fluorescein working solution was added to 20 μL of buffer (blank), Trolox standard solution of various concentrations and serum sample separately. After 20 min of incubation at 37°C, 119 mmol/L ABAP solution freshly prepared in a 20 μL 75 mM phosphate buffer was added to each hole. A multifunctional microplate reader (Infinite M200pro, Tecan Austria GmbH, Salzburg, Austria) was immediately started to continuously measure the fluorescence intensity of each hole and monitor the fluorescence decay at 37°C using an excitation wavelength of 485 nm and an emission wavelength of 538 nm. The measurement was repeated every 4.5 min. Thirty-five cycles were measured (unit: U/mL). One ORAC unit represents the corresponding integral area of 1 μmol/L Trolox on the fluorescence quenching curve.

### Protein expression of the iNOS in the liver

The Western blotting method was used to analyze the protein expression of iNOS. Liver tissue was homogenized in pre-cooled IP lysis buffer with 1 mM PMSF. After standing for 10 min on ice, the homogenate was centrifuged at 12,000 rpm and 4°C for 10 min. The supernatant was then collected. The BCA method was used to determine the protein concentration in the supernatant. Protein loading buffer (5×) was added at a ratio of 4:1. After mixing, the mixture was placed in a boiling water bath for 5 min to cause protein denaturation. The samples were electrophoresed in 10% polyacrylamide gel (SDS–PAGE) (Bio-Rad, USA), and were then transferred to a polyvinylidene fluoride (PVDF) membrane. The non-specific antigens on the membranes were sealed for 2 h. The electrophoresis strips were then incubated with iNOS (1:1000) and β-actin (1:2000) antibodies at room temperature for 4 h. The electrophoresis strips were washed three times with tris-buffered saline and Tween 20 (TBST), followed by incubation with goat anti-rabbit IgG (1:2000) at room temperature for 1.5 h and washed three times with TBST. Finally, the ECL kit was used for chemiluminescence and development. Quantity One image analysis software was used for the analysis.

### Evaluation of mitochondrial structure and function

Mitochondria were extracted from fresh liver tissue according to the instructions for the kit and used to prepare a mitochondrion solution with a suitable concentration. BCA protein assay kit was used to determine the protein content on the multifunctional microplate reader. The mitochondria were stained according to the instructions for the JC-B staining kit, and the integrity of the mitochondria was observed under an optical microscope with an oil lens (CX22, Olympus, Japan). The determination of the mitochondrial membrane potential was made according to the instructions for the mitochondrial membrane potential assay kit with JC-1. JC-1 is a fluorescence probe that accumulates in a mitochondrial matrix to form a polymer that yields red fluorescence; the fluorescence intensity is measured at excitation and emission wavelengths of 525 and 590 nm, respectively. When the membrane potential is relatively low, JC-1 cannot accumulate in a mitochondrial matrix; instead, it exists as a monomer that yields green fluorescence. The fluorescence intensity is thus measured by multifunctional microplate reader at excitation and emission wavelengths of 490 and 530 nm, respectively. The relative ratio of red fluorescence to green fluorescence is used to evaluate the mitochondrial membrane potential. The colorimetric method was used to determine the activities of mitochondrial respiratory chain complexes I and II according to the instructions for the kit.

### Data statistics and analysis

Data are expressed as the means ± standard deviations. The experimental data were processed in Excel. The one-way analysis of variance (ANOVA) mode in the SPSS v17.0 software was used to perform a one-way analysis of variance. The least significance difference (LSD) method and Dunnett’s test were used to test the significance of the results.

## Results

### Mice’s hepatic pathological structure and liver enzyme activity

#### Histopathological observation of mice liver

**Figure 1.**
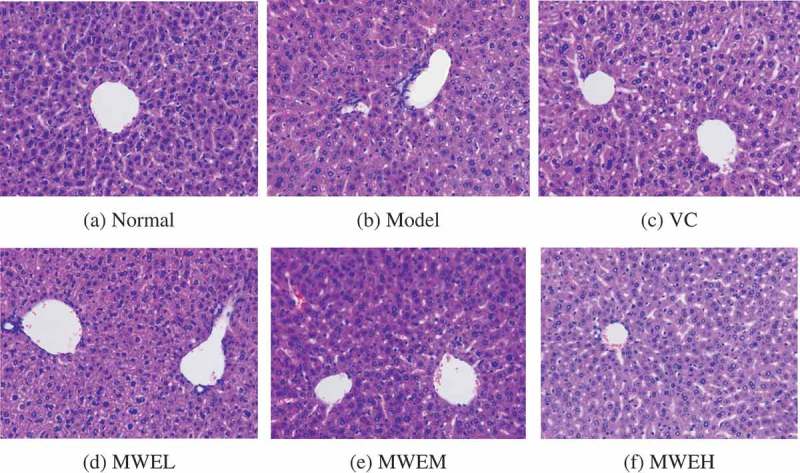
Representative photomicrographs of liver sections from each group of mice stained with hematoxylin and eosin (H&E) (observation under an optical microscope (200×)). (A) NC group: the structure of the liver cells was intact, and the structure of the hepatic cords was clear. (B) Model group: the volume of the liver cells decreased; some individual cells were pyknotic and spottily necrotic. (C) VC group: the structure of the liver cells was basically intact. (D) MWEL group: the structure of the liver cells was relatively intact; the volumes of a small number of liver cells decreased, and some individual liver cells were necrotic. (E) MWEM group: the structure of the liver cells was relatively intact; no significant injury occurred to the liver cells. (F) MWEH group: the structure of the liver cells was intact and in an orderly arrangement, similar to that of the liver cells in the NC group. NC, normal control; Model, restraint stress model; VC, restraint-stressed mice treated with vitamin C 250 mg/kg bw; MWEL, restraint-stressed mice treated with *M. charantia* water extract 250 mg/kg bw; MWEM, restraint-stressed mice treated with *M. charantia* water extract 500 mg/kg bw; MWEH, restraint-stressed mice treated with *M. charantia* water extract 750 mg/kg bw.

Figure 1 shows that the structure of the liver cells in the NC group was intact, and the hepatic cords were clear. The volume of the liver cells in the Model group decreased, and the cytoplasm was stained red; the nuclei of the liver cells were pyknotic and detached. Some individual cells were fragmented and spottily necrotic. The structure of the liver cells in the positive control VC group and various MWE groups was intact; in particular, the structure of the hepatic cords in the MWEM and MWEH group was clear and in an orderly arrangement, indicating that MWE had a certain protective effect on the liver structure.

#### AST and ALT activity in serum

**Figure 2.**
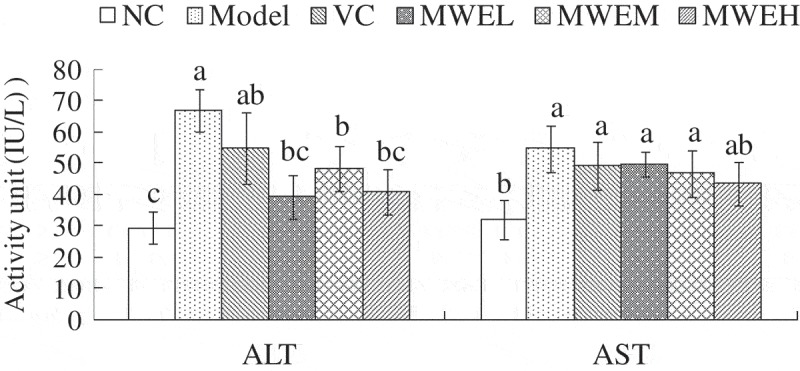
Effect of *Momordica charantia* water extract on the serum ALT and AST activities in the restraint-stressed mice. Values are expressed as the mean ± *SD* (*n* = 10). Different letters for the same index represent significant differences at *p* < .05. NC, normal control; Model, restraint stress model; VC, restraint-stressed mice treated with vitamin C 250 mg/kg bw; MWEL, restraint-stressed mice treated with *M. charantia* water extract 250 mg/kg bw; MWEM, restraint-stressed mice treated with *M. charantia* water extract 500 mg/kg bw; MWEH, restraint-stressed mice treated with *M. charantia* water extract 750 mg/kg bw.

Figure 2 presents the ALT and AST activities in different groups. Figure 2 shows that the ALT and AST activities in the Model group were significantly higher than those in the NC group (*p *< .05), indicating that restraint stress caused hepatocyte lesions in the mouse livers. There was no significant difference between the mice in the positive control VC group and the Model group regarding the activity of ALT, whereas the ALT activity in the MWE groups was significantly lower than that in the Model group (*p *< .05). Compared with the Model group, the AST activity in the positive control VC and MWE groups decreased, but not significantly (*p *> .05). The results indicated that MWE can attenuate ALT activity with no effect on AST in restraint stress mice.

### Antioxidant status in mice

#### GSH content in the liver tissues

Table 2 lists the content of GSH in the different groups. Compared with the NC group, the GSH content in the Model group decreased significantly (*p *< .05), indicating that the restraint stress treatment reduced the oxygen-free-radical scavenging ability of the mouse liver. The GSH content in the positive control VC group increased significantly to the normal level (*p *< .05). The GSH contents in the MWE groups all increased. There was no significant difference between the MWE group and the NC group (*p *> .05).

**Table 2.** Effect of *Momordica charantia* water extract on the redox status in the liver and serum of restraint-stressed mice.

Groups	Liver				Serum	
	GSH (mg/g prot)	GSH-PX (U^#^/mg prot)	TBARS (nmol MDA equivalents/mg prot)	ORAC (U^﹡^/mL)	TBARS (nmol MDA equivalents/mL)	ORAC (U^﹡^/mL)
NC	1.85 ± 0.26^ab^	845.98 ± 54.89^a^	8.60 ± 0.17^cd^	45,251.28 ± 482.79^a^	21.90 ± 0.83^b^	212,727.87 ± 32,807.35^a^
Model	1.31 ± 0.24^c^	512.54 ± 71.53^b^	17.97 ± 1.77^a^	35,139.14 ± 7826.71^b^	27.41 ± 4.25^a^	137,634.14 ± 4885.29^b^
VC	2.28 ± 0.27^a^	828.77 ± 153.04^a^	9.93 ± 2.59^cd^	44,234.03 ± 3609.27^a^	18.94 ± 2.37^bc^	204,685.80 ± 13,517.46^a^
MWEL	1.47 ± 0.25^bc^	563.44 ± 132.25^b^	13.54 ± 1.14^b^	52,899.01 ± 6054.38^a^	20.27 ± 3.14^bc^	202,293.78 ± 39,726.58^a^
MWEM	1.97 ± 0.30^a^	535.65 ± 98.59^b^	8.02 ± 0.97^d^	48,718.48 ± 5602.71^a^	15.84 ± 2.01^c^	208,096.94 ± 44,471.94^a^
MWEH	1.84 ± 0.50^ab^	541.01 ± 88.76^b^	11.15 ± 2.39^bc^	45,619.79 ± 8724.11^a^	18.36 ± 1.57^bc^	200,251.61 ± 21,154.44^a^

U^#^ = One milligram protein decreases 1 μmol/L GSH concentration in the system every minute when the non-enzymatic reaction effect is deducted. U^﹡^ = the corresponding integral area of 1 μmol/L Trolox on the fluorescence quenching curve. Values are expressed as the mean ± *SD* (*n* = 10). The values within each column marked by different letters are significantly different (*p* < .05).

Abbreviations: NC, normal control; Model, restraint stress model; VC, restraint-stressed mice treated with vitamin C 250 mg/kg bw; MWEL, restraint-stressed mice treated with *M. charantia* water extract 250 mg/kg bw. MWEM, restraint-stressed mice treated with *M. charantia* water extract 500 mg/kg bw; MWEH, restraint-stressed mice treated with *M. charantia* water extract 750 mg/kg bw.

#### Activity of GSH-PX in the liver tissues

Table 2 summarizes the GSH-PX activity in the livers of the mice in different groups. Compared with the NC group, the GSH-PX activity in the Model group decreased significantly (*p *< .05), indicating that the restraint stress treatment reduced the H_2_O_2_ scavenging capacity of the mouse livers. The GSH-PX activity in the positive control VC group increased significantly to the normal level. Compared with the Model group, the GSH-PX activities in the MWE groups all increased slightly, but the difference from the Model group did not reach the level of significance (*p *> .05), indicating that MWE had an insignificant effect on the H_2_O_2_ scavenging capacity of the liver.

#### Lipid peroxide content in the liver tissues and serum

Table 2 lists the TBARS contents in the livers and sera of the mice in different groups. The TBARS content in the livers of the Model group was significantly higher than that of the NC group (*p *< .05), indicating that restraint stress can cause the production of large amounts of lipid peroxides in mouse livers. Compared with the Model group, the TBARS contents in the positive control VC group and the MWE groups all decreased significantly (*p *< .05); in addition, the accumulations of TBARS in the MWEM and MWEH groups all decreased to the normal level, indicating that MWE can significantly improve the liver antioxidant status.

The serum TBARS content in the mice in the Model group was significantly higher than that in the NC group (*p *< .05). Compared with the Model group, the TBARS contents in the positive control VC group and the MWE groups all decreased significantly (*p *< .05), and there was no significant difference among these four groups; in addition, the TBARS content in the MWEM group was the lowest. MWE reduced the TBARS content to the normal or below-normal level, indicating that MWE can significantly improve the antioxidant status in the body.

#### ORAC values of the mouse serum and liver

Table 2 lists the ORAC values of the serum and liver in the different groups; the trends of change in the ORAC values of the serum and liver were the same. Compared with the NC group, the ORAC values of the liver and serum in the Model group decreased significantly (*p *< .05), indicating that restraint stress reduced the ORAC values of the liver and body. Compared with the Model group, the ORAC values of the liver and serum in the positive control VC group and MWE groups all increased significantly (*p *< .05); there was no significant difference among the MWE groups, and they could all reach the level of the NC group (*p *> .05), indicating that MWE significantly increased the ORAC values of the liver and body.

#### NO content, activity and protein expression level of iNOS in the mouse livers

Table 3 lists the NO content and activities of iNOS in the mouse livers from the different groups. The NO content and activity of iNOS in the Model group was significantly higher than that in the NC group (*p *< .05). Compared with the Model group, the NO contents in the positive control VC group and the MWE groups all decreased significantly (*p *< .05). There was no significant difference among the MWE groups and VC group. The iNOS activity in the positive control VC group decreased significantly but was still higher than that in the NC group (*p *< .05). Compared with the Model group, the iNOS activities in the MWE groups all decreased significantly (*p *< .05) and reached the level of the NC group (*p *> .05), indicating that MWE could significantly decrease the enzyme activities caused by restraint stress, which reduced the production of NO.

**Table 3.** Effect of *Momordica charantia* water extract on inflammatory cytokines in the livers of restraint-stressed mice.

Groups	NO (μmol/g prot)	iNOS (U^#^/mg prot)
NC	0.25 ± 0.10^d^	1.92 ± 0.16^c^
Model	0.76 ± 0.17^a^	4.20 ± 0.58^a^
VC	0.42 ± 0.09^bc^	3.03 ± 0.82^b^
MWEL	0.32 ± 0.08^cd^	1.69 ± 0.37^c^
MWEM	0.51 ± 0.04^bc^	1.39 ± 0.23^c^
MWEH	0.53 ± 0.05^b^	1.72 ± 0.56^c^

U^#^ = every mg of tissue protein that produces 1 nmol of NO every minute. Values are expressed as the mean ± *SD* (*n* = 10). The values within each column marked by different letters are significantly different (*p* < .05). Abbreviations: NC, normal control; Model, restraint stress model; VC, restraint-stressed mice treated with vitamin C 250 mg/kg bw; MWEL, restraint-stressed mice treated with *M. charantia* water extract 250 mg/kg bw.MWEM, restraint-stressed mice treated with *M. charantia* water extract 500 mg/kg bw; MWEH, restraint-stressed mice treated with *M. charantia* water extract 750 mg/kg bw.

**Figure 3.**
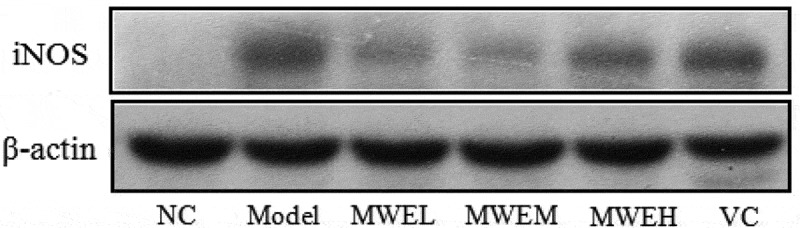
Effect of *Momordica charantia* water extract on iNOS expression in the livers of restraint-stressed mice. NC, normal control; Model, restraint stress model; VC, restraint-stressed mice treated with vitamin C 250 mg/kg bw; MWEL, restraint-stressed mice treated with *M. charantia* water extract 250 mg/kg bw; MWEM, restraint-stressed mice treated with *M. charantia* water extract 500 mg/kg bw; MWEH, restraint-stressed mice treated with *M. charantia* water extract 750 mg/kg bw.

Figure 3 reveals that the iNOS protein expression level in the mouse livers from the Model group was significantly higher than that in the NC group (*p *< .05). Compared with the Model group, the iNOS expression levels in the positive control VC group and the MWE groups decreased significantly (*p *< .05); in addition, the improving effect of MWE was significant in the MWEM groups, indicating that MWE could significantly reduce the iNOS expression level and reduce the corresponding enzyme activity.

### Mitochondrial structure and function of the mouse livers

#### ROS content in the mouse liver mitochondria

Table 4 lists the ROS content in the mouse livers. Compared with the NC group, the ROS content in the Model group increased significantly (*p *< .05). The ROS content in the positive control VC group decreased significantly to the normal level (*p *> .05). However, the ROS contents in the MWE groups were all significantly lower than that in the Model group (*p *< .05), and there was no significant difference among the MWE groups (*p *> .05).

**Table 4.** Effect of *Momordica charantia* water extract on ROS, MMP (JC-1 ration) and the mitochondrial respiratory chain complex I and II activities in the livers of restraint-stressed mice.

Groups	ROS (relative fluorescence intensity)	Membrane potential(aggregate/monomer)	Complex I(μmol/mg prot)			Complex II(μmol/mg prot)		
			1 min	2 min	3 min	1 min	3 min	5 min
NC	1974.75 ± 477.50^cd^	0.98 ± 0.01^a^	9.60 ± 1.02^a^	24.29 ± 2.78^a^	43.34 ± 7.20^a^	41.09 ± 7.34^a^	47.26 ± 8.78^a^	49.27 ± 5.04^a^
Model	4888.83 ± 448.27^a^	0.71 ± 0.08^c^	4.41 ± 0.48^d^	15.25 ± 4.35^c^	22.95 ± 2.95^b^	10.38 ± 6.23^c^	13.52 ± 7.52^c^	17.07 ± 6.91^d^
VC	1555.17 ± 206.05^d^	0.82 ± 0.10^b^	7.93 ± 1.67^b^	21.12 ± 2.72^ab^	36.45 ± 1.51^a^	19.96 ± 3.31^b^	23.09 ± 4.99^b^	27.98 ± 4.20^c^
MWEL	2883.17 ± 431.78^bc^	0.82 ± 0.09^b^	6.25 ± 0.15^c^	18.39 ± 1.08^bc^	28.89 ± 5.82^b^	27.32 ± 8.92^b^	30.61 ± 9.32^b^	38.12 ± 8.51^b^
MWEM	2963.83 ± 692.56^bc^	0.83 ± 0.05^b^	7.66 ± 0.47^c^	20.82 ± 0.24^ab^	37.90 ± 2.31^a^	22.85 ± 6.41^b^	26.41 ± 4.43^b^	32.57 ± 5.39^bc^
MWEH	3452.51 ± 956.17^b^	0.84 ± 0.05^b^	8.72 ± 0.17^ab^	21.72 ± 1.71^ab^	37.17 ± 1.45^a^	25.42 ± 6.08^b^	30.81 ± 7.83^b^	34.16 ± 8.61^bc^

Values are expressed as the mean ± *SD* (*n* = 8). The values within each column marked by different letters are significantly different (*p* < .05). Abbreviations: NC, normal control; Model, restraint stress model; VC, restraint-stressed mice treated with vitamin C 250 mg/kg bw; MWEL, restraint-stressed mice treated with *M. charantia* water extract 250 mg/kg bw; MWEM, restraint-stressed mice treated with *M. charantia* water extract 500 mg/kg bw; MWEH, restraint-stressed mice treated with *M. charantia* water extract 750 mg/kg bw.

#### Mitochondrial membrane potential in the mouse livers

Table 4 lists the changes in the mitochondrial membrane potential in the mouse livers. Compared with the mice in the NC group, the membrane potential in the Model group was significantly lower (*p *< .05). The mitochondrial membrane potentials in the positive control VC group and the MWE groups all increased significantly (*p *< .05). There was no significant difference among the MWE groups, indicating that MWE could protect the integrity of the mitochondrial membrane.

#### Activities of the mitochondrial respiratory chain complexes in the mouse livers

Table 4 lists the activities of mitochondrial respiratory chain complexes I and II in the mouse livers during the 1^st^, 2^nd^ and 3^rd^ minutes. The activities of complex I in the Model group during the various time periods were significantly lower than those in the NC group (*p *< .05). The activity of complex I in the positive control VC group increased significantly and reached the normal level during the 2^nd^ and 3^rd^ minutes (*p *> .05). Compared with the Model group, the activities of complex I in the MWE groups all increased; the activities of complex I in the MWEM and MWEH groups all reached the normal level during the 2^nd^ and 3^rd^ minutes (*p *> .05).

Compared with the NC group, the activity of complex II in the Model group decreased significantly during the various time periods (*p *< .05). The activity of complex II in the positive control VC group increased significantly but was still lower than that in the NC group (*p *< .05). Compared with the Model group, the activities of complex II in the MWE groups all increased significantly (*p *< .05), but there was no significant difference among the MWE groups (*p *> .05), suggesting that MWE could counteract the decreases in the activity of complexes I and II caused by the restraint stress.

## Discussion

### Effect of MWE on the anti-oxidative capacity of restraint-stressed mice

During the restraint stress process, the production and scavenging of ROS are unbalanced; the excessive free radicals then react with the proteins, lipids and nucleic acids in the body. In addition, oxygen free radicals attack the unsaturated fatty acids of the biofilm, resulting in lipid peroxidation and destroying the integrity of the cell membrane structure [20]. Kurihara et al. claimed that the liver is the primary organ of metabolism and is easily attacked, resulting in membranolysis and liver injury in mice treated with restraint stress [21]. The present investigation revealed that restraint stress caused the ALT and AST activities to increase significantly, which is in agreement with the results from the studies conducted by Li et al. and Kurihara et al. [7,21]. MWE reduced the activities of ALT with no effect on AST in restraint stress mice.

Oxidative stress is one of the main mechanisms of liver injury. The present study demonstrated that the GSH content and activities of GSH-PX in the bodies of the restraint-stressed mice all decreased, and the lipid peroxide content in these mice was significantly higher than that in the NC group; in addition, the total ORAC values of the restraint-stressed mice decreased significantly. MWE enhanced the anti-oxidative capacity of the mouse livers by initiating the enzymatic and non-enzymatic protective systems existing in the body. Furthermore, MWE reduced the lipid peroxidation of polyunsaturated fatty acids caused by free radicals and the production of the final product, TBARS, to protect the body against oxidative damage. The study conducted by Thenmozhi et al. showed that *M. charantia* fruit aqueous extract can reduce the ALT and AST activities and the TBARS content, increase the GSH content, and increase the GSH-PX and catalase (CAT) activities in hyperammonemic rats, indicating that *M. charantia* aqueous extract can increase the enzyme activity and enhance the antioxidant capacity of the body [22]. This may be attributed to the presence of higher amounts of phenolics and flavonoids, which have been reported as potential antioxidants [4,23,24]. Furthermore, polysaccharide from *M. charantia* showed potentials in antioxidant properties in vitro and vivo [13,25].

Additionally, in this study, the level of NO in MWE treated mice live was minimized, which might possibly be due to the inhibition of iNOS protein expression by MWE. NO plays a paradoxical role in liver physiology. Small amounts of NO induced by endothelial nitric oxide synthase (eNOS) have a cytoprotective effect; while overproduction of NO induced by iNOS may be cytotoxicity to liver; Peroxynitrite (ONOO^−^) is the byproduct of NO, which also causes further hepatic injury due to its potent oxidative effect [26]. *M. charantia* polysaccharides were reported had direct scavenging effects on NO, O_2_^−^and ONOO^−^ [27]. Moreover, Jain et al. found *M. charantia* fruit extract (including >8% bitters: momordicosides K [3%] and L [2%], and momordicines I [2%] and II [3%]; gallic acid 6%) protect against vincristine induced neuropathic pain in rats by modulating NOS inhibition and antioxidative activity [28]. 5*β*,19-epoxy-25-methoxy-cucurbita-6,23-diene-3*β*,19-diol, a triterpene purified from *M. charantia* was found to suppress the expression of iNOS in FL83B hepatocyte cells [29]. These finding suggested saponin involved the inhibition of NOS. Furthermore, gallic acid inhibits iNOS in stressed and unstressed mice [30]. Although we did not measure the gallic acid content in the study, gallic acid was the principal compound of boiling water extract of *M. charantia* fruit [24].

#### Effect of MWE on the mitochondrial structure and function in the livers of restraint-stressed mice

Mitochondria are the main sites where ROS (including O_2_^−^, OH^−^ and H_2_O_2_) are produced and are also the sites for cell energy conversion. When dysfunction occurs in the body, H^+^ flows out of the respiratory chain, forming a negative transmembrane potential inside the membrane relative to the outside. During this process, electron leakage will occur, causing the reduction of O_2_ to O_2_^−^ and the conversion of partial O_2_^−^ to H_2_O_2_, which further converts to OH^−^, in turn giving rise to the excessive production of mitochondrial ROS and the occurrence of a series of injuries [31]. It was discovered in the present study that restraint stress could increase the mitochondrial ROS content, which further proved that oxidative stress injury was one of the mechanisms of restraint stress.

As the ROS content increases, mitochondria become the main sites attacked by the ROS [32]. Iqbal and Hood and Singh et al. have reported that oxidative stress can reduce the mitochondrial membrane potential, resulting in apoptosis, which ultimately affects the corresponding tissue function [33,34]. With the decreasing mitochondrial membrane potential, membrane phospholipids and proteins are damaged, resulting in mitochondrial structural damage. The decreased activities of mitochondrial complexes and the impaired function of oxidative phosphorylation cause an increased production of ROS once again [35,36]. The present study revealed that after the mice underwent the restraint stress treatment, both the mitochondrial membrane potential and the activities of respiratory chain complexes I and II decreased, which was in agreement with the findings of Yao et al. [37]. However, MWE can reduce the mitochondrial ROS content and thus prevent the opening of mitochondrial permeability transition pores, effectively increasing the transmembrane potential, maintaining the activities of respiratory chain complexes I and II, and reducing oxygen consumption, which consequently reduces the production of apoptosis factors and prevents apoptosis. Similar findings were reported by Jain et al. They pointed out that the protective effect of *M. charantia* on mitochondria could be due to the presence of polyphenols and saponins, as these compounds have been shown to maintain the mitochondrial respiratory complexes [28]. Saponins protect mitochondria through activate CaMKKβ-AMPK, in a calcium-independent manner [38]. Polyphenols possess a strong free radical-scavenging capacity and can protect mitochondria against free radicals by increasing the oxidative phosphorylation efficiency and mitochondrial respiratory chain electron transport speed [39].

### Use of MWE for management injianghuo effect

Some research suggests that the beneficial role of natural products and herbal medicine in various disease conditions was strengthened by the synergy effect of chemicals present in it [40]. For example, antinociceptive actions of *M. charantia* fruit extract (gallic acid 6% and >8% bitters) was better than its marker compound gallic acid [28]. MWE used in the present study was a mixture of polysaccharide, saponin, protein and phenolic, which might have pharmacological actions. Thus, each constituent might affect different targets in the protective effect against liver injury in restraint-stressed mice of MWE, and the synergistic action of these constituents would result in superior effects of MWE than by a single constituent.

Considering the results and the properties of MWE described here, the effective dose is specified as 500 mg/kg. The effect of 500 mg/kg of MWE was equivalent to 250 mg/kg VC. Taking into account the average body weight of 60 kg, the 10-fold faster metabolism of mice than humans in general, the daily intake of 500 mg/kg MWE for a mouse corresponds to a human consumption of approximately 3 g of MWE per day. Assuming an average yield rate of MWE is 43%, a 60-kg man would need to drink approximately 7.5 g *M. charantia* dried fruit to get an equivalent dose. Our studies would suggest that this level of consumption is effective to improve hepatic oxidative stress defense systems. Moreover, long-term regular modest *M. charantia* consumption may have additional health benefits not addressed here.

## Conclusions

The present study showed that MWE has a significant protective effect against liver injury in restraint-stressed mice. The mechanism of the protective effect of MWE may be summarized in two aspects: (1) enhancing the anti-oxidative capacity of restraint-stressed mice by increasing GSH-PX activity and GSH content in the livers and reducing the overproduction of the lipid peroxidation product and NO in the liver and the whole body; (2) protecting liver mitochondrion by reducing the production of mitochondrial ROS, restoring the mitochondrial membrane potential, and enhancing the activities of respiratory chain complexes I and II. The results of the present investigation provide important bases for revealing the bioactivity of *M. charantia* and are significant for guiding the development of *M. charantia* functional foods.

## Supplementary Material

supll-renamed_2020d.doc
