# Supplementary material for: Protective effect of Momordica charantia water extract against liver injury in restraint-stressed mice and the underlying mechanism
Source: Food Nutr Res. 2017 Jul 13;61(1):1348864. doi: 10.1080/16546628.2017.1348864 (PMC5510204; doi:10.1080/16546628.2017.1348864)
Supplement: supll-renamed_2020d.doc [file zfnr_a_1348864_sm7326.doc]

Supplementary Fig.1 structure of triterpenoid saponins standard samples

Supplementary Table 1.

Main monosaccharide composition（Molar ratio）of polysaccharide and amino acid composition of protein in *Momordica charantia* water extract.

|  | Compounds | Content |
| --- | --- | --- |
| Polysaccharide composition（Molar ratio） | Ribose | 5.35 |
|  | Rhamnose | 1.00 |
|  | Arabinose | 36.91 |
|  | Xylose | 20.34 |
|  | Mannose | 4.03 |
|  | Glucose | 11.75 |
|  | Galactose | 369.76 |
| Protein composition ^a^  (g/100g) | Alanine | 0.38±0.02 |
|  | Arginine | 2.15±0.11 |
|  | Aspartic acid | 0.65±0.05 |
|  | Glutamic acid | 1.16±0.09 |
|  | Glycine | 0.24±0.01 |
|  | Histidine | 0.31±0.03 |
|  | Isoleucine | 0.24±0.02 |
|  | Leucine | 0.36±0.03 |
|  | Lysine | 0.50±0.08 |
|  | Methionine | 0.14±0.01 |
|  | Phenylalanine | 0.54±0.01 |
|  | Proline | 0.64±0.02 |
|  | Serine | 0.45±0.02 |
|  | Threonine | 0.50±0.03 |
|  | P-hydroxyphenylalanine | 0.23±0.01 |
|  | Valine | 0.59±0.05 |

^a^ Values are the means± SD (n = 3).
